# Assessment of the phenolic compounds of pearl and finger millets obtained from South Africa and Zimbabwe

**DOI:** 10.1002/fsn3.1778

**Published:** 2020-08-15

**Authors:** Zahra Mohammed Hassan, Nthabiseng Amenda Sebola, Monnye Mabelebele

**Affiliations:** ^1^ Department of Agriculture and Animal Health College of Agriculture and Environmental Sciences University of South Africa Pretoria South Africa

**Keywords:** flavonoids, high‐resolution UPLC‐MS, millet, phenolic acids, phenolic compounds

## Abstract

Millet grains are rich in phenolic compounds which have health benefits. This study aims to elucidate the phenolic properties of pearl and finger millet grown in South Africa and Zimbabwe. The milled samples were analyzed by Waters Synapt G2 Quadrupole time‐of‐flight (QTOF) mass spectrometer (MS). A total of eight phenolic compounds were detected and quantified in the millet varieties, which included derivatives of benzoic acid such as protocatechuic and p‐hydroxybenzoic acids. Flavonoids such as catechin, epicatechin, procyanidin B1, procyanidin B2, and kaempferol glycoside were also detected. Generally, catechin was the dominant phenolic compound, followed by epicatechin. The mean values for catechin ranged from 2.50 to 12.6 mg/kg for the pearl millet and 610.4 to 675.1 mg/kg for the finger millet. While the epicatechin mean values ranged between 1.2 to 1.8 for pearl millet and 99.1 to 139.5 for finger millet. Procyanidins B1 and B2 were only detected and quantified in the finger millet types, While Kaempferol glycoside was only recorded in the pearl millets with mean values of 196.0 mg/kg for pearl millet South Africa and 213.6 mg/kg for pearl millet Zimbabwe. There was a difference among the varieties for the content of kaempferol glycoside. Protocatechuic and p‐hydroxybenzoic acids were only present in the finger millet types, their mean values were (20.9, 23.7 mg/kg) and (16.8,13.5 mg/kg) respectively. It can be substantiated from the outcome of this study that millet can be used as a source of valuable phenolic compounds and that the variety of millet is the determining factor of the phenolic compound content.

## Graphical abstract

Phenolic compounds in millets.

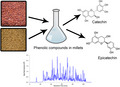

## 1 INTRODUCTION

Millet is regarded as one of the economically important cereals after maize, rice, wheat, barley, and sorghum (Prasad & Staggenborg, 2009). It is mostly grown in semi‐arid zones and is used for human food and livestock feed (Amadou, Le, Amza, Sun, & Shi, 2013). Nutritionally, millet is on par with other popular cereals and its grains are abundant in phytochemicals making it very vital as feed and food (Chandrasekara, Naczk, & Shahidi, 2012; Shahidi & Chandrasekara, 2013). Furthermore, they are natural sources of antioxidants in food and biological systems which have health benefits that may work against several pathophysiological conditions (Shahidi & Chandrasekara, 2013). Among the physiognomies of millet which need to be explored are the phenolic compounds.

Phenolic compounds are a class of secondary metabolites found in plants and further divided into phenolic acids and polyphenols (Giada, 2013). Furthermore, phenolic acids are divided into two classes: hydroxybenzoic and hydroxycinnamic acids (Dykes & Rooney, 2006). The main dietary phenolic compounds include the phenolic acids, flavonoids, and tannins (King & Young, 1999). The phenolic acids and flavonoids are also considered vital in promoting health by reducing the risk of metabolic syndrome and the related complications of type 2 diabetes (Lin et al., 2010). To date, there is no uniform set of identified and quantified phenolic compounds in different millet varieties.

Chethan, Sreerama, and Malleshi (2008) identified nine phenolic acids which include gallic acid, protocatechuic acid, p‐hydroxybenzoic acid, vanillic acid, ferulic acid, syringic acid, trans‐cinnamic acid, and p‐coumaric acid, in millets. On the other hand, Chandrasekara and Shahidi, (2011) found that hydroxycinnamic acids and their derivatives were the main contributors to the total phenolic compounds of insoluble‐bound phenolic fraction of millet varieties. However, in another study by Xiang, Apea‐Bah, Ndolo, Katundu, and Beta (2019), flavonoids were found to be the predominant phenolic compound in different millet varieties, whereas Sharma, Sharma, Handa, and Pathania (2017) reported higher amounts of phenolic content and antioxidant activity in methanolic extracts of kodo millet grains. In like manner, Pradeep and Sreerama (2015) found that kaempferol was the most abundant flavonoid in raw millet varieties. In comparison, the most dominant phenolic compounds in maize varieties are phenolic acids followed by flavonoids (García‐Salinas, García‐Salinas, Alemán‐de la Torre, & Ramírez‐Díaz, 2017). It is, however, important to realize that different analysis methods also affect the total phenolic compound contents of plants. Chethan and Malleshi (2007) argue that although different solvents are being used to extract phenolic compounds from plant foods, acidified methanol is the best organic solvent for extraction of phenolic compounds from millets. Other factors such as environmental conditions, cultivar of a plant, processing conditions, and storage are also found to affect the quantity of phenolic compounds in plants. Environmental factors such as sun exposure, soil type, and rainfall influence the phenolic content of plants (Manach, Augustin, Morand, Remesy, & Jimenez, 2004). Similarly, Shahidi and Naczk (2004) also reported that the type and the content of the phenolic compounds of grains depend on the type of millet, variety, part of the grain, climatic conditions, and cultivation practices.

More studies to expansively profile the phenolic compounds of millet types in different locations are therefore warranted. With all these interesting incites and the importance of millet grains mentioned above, studies on phenolic compounds in millet are limited, particularly in the Southern African region. This study aims to investigate the phenolic compounds of pearl and finger millet grains obtained from South Africa and Zimbabwe by LC‐MS method using the Synapt G2 qTOF from Waters (Milford, USA).

## 2 MATERIALS AND METHODS

### 2.1 Plant materials

Pearl millet (*Pennisetum glaucum*) used in this study was obtained from ARC‐Grain Crops Institute in Potchefstroom, in the North West province of South Africa. The variety, ARC‐PM 01, was imported from ICRISAT‐Bulawayo in 2005, among other pearl millet germplasm, for performance test under South African conditions. After testing over three locations in South Africa (Potchefstroom, Taung, Polokwane) over two seasons, the variety was selected as top performer among 36 varieties based on agronomic desirability and grain yield. Variety ARC‐PM 01 further went under e regress selection over the years through conventional breeding and submitted for variety listing for production in SA. The variety was grown during 2017/18 growing season at the ARC‐Grain Crops Experimental Farm, Potchefstroom, North West Province. The finger millet (*Elusine coracana*) was sourced from a local market in Johannesburg. The Zimbabwean millet samples were obtained from local market in Harare, Zimbabwe. The samples were milled using a grinder and sifted to produce a fine texture. The samples were prepared in duplicate and ready for further analysis.

### 2.2 Extraction of phenolic compounds

The extracts were prepared by using 2 g dry millet material + 15 ml 50% methanol/1% formic acid in water with ultrasonication for 1 hr and standing overnight, followed by centrifugation and transfer of the supernatant to a glass vial ready for the LC‐MS analysis.

### 2.3 LC‐MS analysis

The samples were analyzed by LC/MS quadrupole time‐of‐flight (QTOF) mass spectrometer (MS) connected to a Waters Acquity ultra‐performance liquid chromatograph (UPLC) (Waters, Milford, MA, USA) was used for high‐resolution UPLC‐MS analysis. Electrospray ionization was used in negative mode with a cone voltage of 15 V, desolvation temperature of 275°C, desolvation gas at 650 L/h, and the rest of the MS settings optimized for best resolution and sensitivity. Data were acquired by scanning from 150 to 1,500 *m/z* in resolution mode as well as in *MSE* mode. In *MSE* mode, two channels of MS data were acquired, one at a low collision energy (4 V) and the second using a collision energy ramp (20−60 V) to obtain fragmentation data as well. Leucine enkephalin was used as lock mass (reference mass) for accurate mass determination, and the instrument was calibrated with sodium formate. Separation was achieved on a Waters HSS T3, 2.1 × 100 mm, 1.7 μm column. An injection volume of 2 μL was used, and the mobile phase consisted of 0.1% formic acid (solvent A) and acetonitrile containing 0.1% formic acid as solvent B. The gradient started at 100% solvent A for 1 min and changed to 28% B over 22 min in a linear way. It then went to 40% B over 50 s and a wash step of 1.5 min at 100% B, followed by re‐equilibration to initial conditions for 4 min. The flow rate was 0.3 ml/min, and the column temperature was maintained at 55°C.

### 2.4 Statistical analysis

Data obtained were subjected to a one‐way analysis of variance of the statistical analysis software (SAS, 2010). Where mean values were statistically significant at *p* < .05, Duncan's multiple range test was used. The Principal component analysis (PCA) analysis was conducted using PAST version 4.02, a software for scientific data analysis, with functions for data manipulation, plotting, univariate and multivariate statistics analysis.

## 3 RESULTS AND DISCUSSION

Table 1 shows the retention time, fragments, elemental composition, and tentative identification of phenolic compounds detected in the four millet varieties. Total of eight (8) phenolic compounds were detected and quantified in the millet types, which included derivatives of benzoic acid such as protocatechuic acid and p‐hydroxybenzoic acid. Flavonoids such as catechin, epicatechin, procyanidin B1, procyanidin B2, and kaempferol glycoside were also detected. Tryptophan was the only amino acid detected in the millet varieties. Citric acid was also detected but not quantified in any of the varieties. Numerous unknown compounds were also detected during the analysis which were later assigned to suggested compounds using the elemental composition, through SCI‐FINDER application. Generally, finger millet varieties had more phenolic compounds than the pearl millet types, irrespective of where they were grown.

**TABLE 1:** Phenolic compounds identified in the extracts of millet grains using LC‐MS methods

No	Retention time	[M‐H]‐formula	Fragments	[M‐H] elemental composition	UV_ma_	Tentative identification
1	1,690	191,018	Nd	C6H7O7	265	Citric acid
2	7,350	153,019	153,109	C7H5O4	258,294	Protocatechuic acid
3	8,540	203,0811	190	C11H11N2O2	278	Tryptophan
4	9.178	137,0235	128,117	C7H5O3	277,320	P‐hydroxybenzoic acid
5	9,820	577,137	289,161,125	C30H25O12	278	Procyanidin B1
6	10,45	289,0673	Nd	C15H13O6	279	Catechin
7	12,46	289,0704	Nd	C15H13O6	279	Epicatechin
8	13,93	577,1281	Nd	C30H25O12	278	Procyanidin B2
9	14,78	609,1454	489,429,327,309,297	C27H29O16	268,347	Kaempferol glycoside

### 3.1 Total flavonoids

**FIGURE 1:**
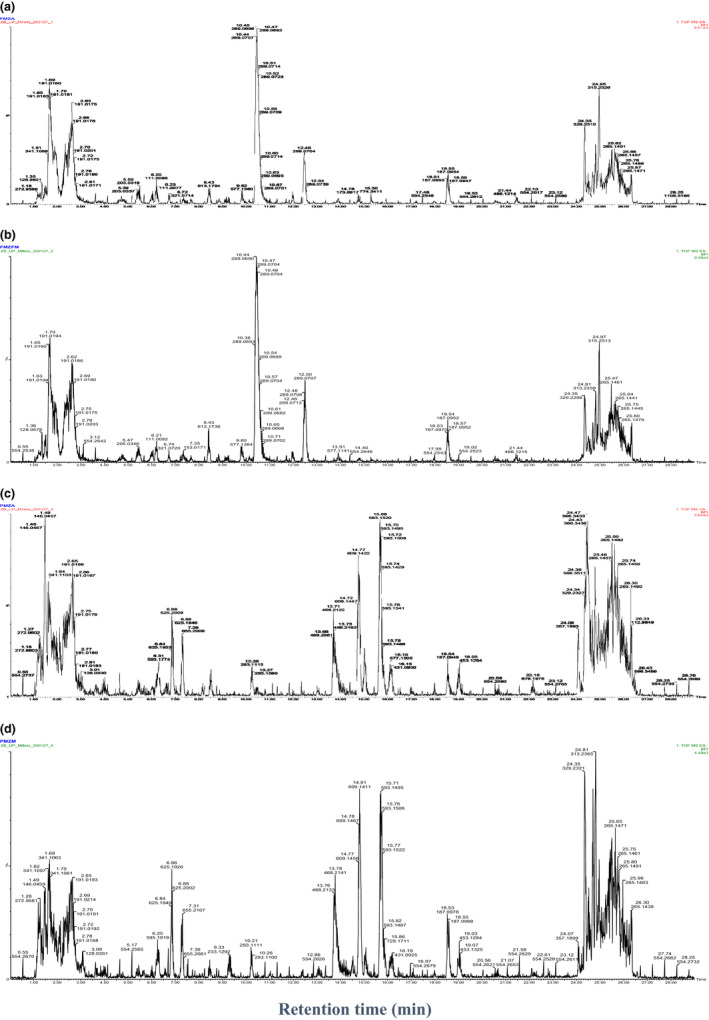
Chromatograms of millet types, (a) finger millet South Africa, (b) finger millet Zimbabwe, (c) pearl millet South Africa, and (d) pearl millet Zimbabwe

The contents of flavonoids in the millet varieties determined by the LC‐MS method are shown in (Table 2). Finger millets were recorded to have higher flavonoids than those found in the pearl millet types, indicating varietal differences. Catechin was the dominant flavonoid (Figure 1a,b) followed by epicatechin. These findings are consistent with Xiang et al. (2019), where catechin and epicatechin were the abundant flavonoids in finger millet varieties. Surprisingly, these findings disagree with the findings of Rao and Muralikrishna, (2002) which states that the phenolic acids and tannins are the main polyphenols in cereals and that flavonoids are present in small quantities. There was a highly significant difference (*p* < .05) in catechin and epicatechin of all the millet varieties. Finger millet varieties had the highest average mean values of 642.75 mg/kg in finger millet South Africa (FMZA) and 119.3 mg/kg in finger millet Zimbabwe (FMZM). In addition, kaempferol glycoside was only detected in the pearl millet types, with mean values of 196.0 in pearl millet and 213.6 mg/kg. The highest value was detected in pearl millet Zimbabwe (PMZM) and lowest in pearl millet South Africa (PMZA). These differences could be attributed to different locations and the place of origin of the millets used in the study.

**TABLE 2:** Quantified individual phenolic compounds in millet varieties (mg/kg)

Samples	FMZA	FMZM	PMZA	PMZM	*SEM*	Probability
Protocatechuic acid	20.90^b^	23.70^a^	Nd	Nd	0.0500	.001
Catechin	610.4^b^	675.1^a^	12.6^c^	2.50^d^	0.0025	.001
Epicatechin	99.10^b^	139.5^a^	1.80^c^	1.20^d^	0.0050	.001
Tryptophan	3.000^a^	2.100^b^	16.5^c^	12.5^d^	0.0500	.001
p‐hydroxybenzoic acid	16.80^a^	13.50^b^	Nd	Nd	0.0354	.001
Procyanidin B1	28.40^b^	31.90^a^	Nd	Nd	0.0354	.001
Procyanidin B2	15.50^b^	15.80^a^	Nd	Nd	0.0354	.001
Kaempferol glycoside	Nd	Nd	196.0^b^	213.6 ^a^	0.0289	.001

Note

Mean values of individual polyphenols. a, b, c, d: Means in the same row with different superscripts are significantly different at (*p* < .05).

Abbreviation: Nd, not detected.

In addition, Chandrasekara and Shahidi (2011b) had reported the presence of procyanidin B1 and procyanidin B2 in finger millet; likewise, this study recorded the presence of procyanidin in the finger millet varieties. The possibilities of occurrence of all of these compounds in the millet genotype are therefore justifiable.

**FIGURE 2:**
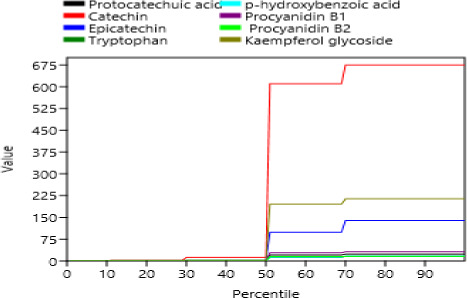
Percentile plot of the phenolic compounds present in the millet varieties

Moreover, it can be seen in Figure 1a that catechin was recorded at 10.45 min retention time and [M‐H] of 289.0673 while epicatechin peaked at retention time of 12.46 min and [M‐H] of 289.0704 for the finger millet type. Consecutives chromatograms of pearl millet types showed no presence of the two compounds but high peaks for the flavonoids kaempferol glucoside at the retention rate of 14.78 and [M‐H] of 609.1454 (Figure 1c,d). Furthermore, the predominance of the catechin in finger millet varieties is clearly indicated by the high peaks and a high percentile as shown in Figures 1 and 2, while p‐hydroxybenzoic acid and procyanidin B2 registered their presence at the bottom of the percentile. Compounds 6 and 7 (catechin and epicatechin) were detected at the same M‐H formula at 289, suggesting that the two compounds are isomers. Likewise, compounds 5 and 8 (procyanidin B1 and procyanidin B2) were detected at the same M‐H formula of 577. In addition, it can also be noted that many peaks with almost similar retention time and M‐H as catechins were captured but not identified, indicating the possibility of other dimers of catechin and epicatechin might exist in the millet types investigated.

### 3.2 Phenolic acids

The first compounds to be identified were citric acid at a retention time of 1.69 min; however, its fragment was not detected, and it was not quantified in any of the millet types, as shown in Table 1. However, p‐hydroxybenzoic acid, which is a monohydroxy benzoic acid, a phenolic derivative of benzoic acid, was quantified but only detected in the finger millet varieties with the mean values of 13.5 and 16.8 mg/kg. The highest mean value was in the finger millet grown in Zimbabwe. Protocatechuic acid, on the other hand, was the predominant phenolic acid found in finger millets ranging from 20.9 to 23.7 mg/kg. There was no presence of this acid detected in the pearl millet types. Both protocatechuic and p‐hydroxybenzoic acids were detected at the retention times of 7.35 and 9.178 and [M‐H] formula of 153.019 and 137.0235. The presence of protocatechuic acid in the varieties investigated indicates possible health benefits, as this acid is known to have pharmaceutical properties, such as antiatherosclerosis, antiviral, antifibrotic, and anticancer (Kakkar & Bais, 2014; Chandrasekara & Shahidi, 2011a). The data presented further substantiate the health benefit associated with finger millet consumption with regard to its flavonoid components. Contrary to the findings by Ralph et al. (2004); Chandrasekara and Shahidi, (2011) who state that hydroxycinnamic acids were the most common phenolics present in the insoluble‐bound millet grains, this study did not record any presence of hydroxycinnamic acids. The most logical explanation would be the methods of analysis used in these studies. Interestingly, during the course of this study, the two most common identified phenolic acids are protocatechuic and p‐hydroxybenzoic acids, and this might indicate the ease of detection of these two acids using different analytical methods.

### 3.3 Principal component analysis (PCA)

**FIGURE 3:**
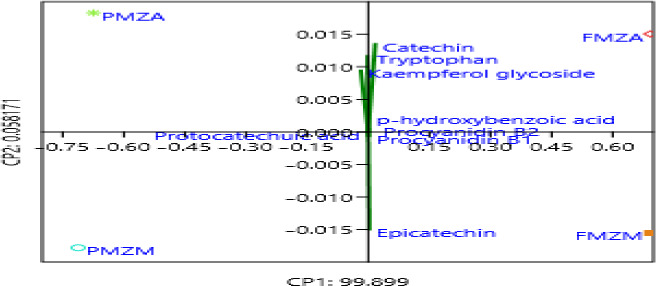
Principal component analysis scatter plot of the phenolic compound

Principal component analysis was carried out using the methods of Hammer, Harper, and Ryan (2001) to further investigate possible variations among the millet varieties. Figure 3 shows a loading of procyanidin B1 and B2, and p‐hydroxybenzoic acid in the same area on the right middle side of the score plot, indicating possible link between these phenolic compounds. In the same way, catechin, tryptophan, and kaempferol glycoside clustered at the right topside of the plot. In the model plotted, the principal components (PCs) explained variance of 99.96% of the data. The two principal components PC1 and PC2 had variabilities of 99.8990 and 0.05817, respectively, as listed in Table 3. The loading plots shown in Figure 2 indicate that a positive correlation exists between principal component 1 (PC1) and catechin, epicatechin, p‐hydroxybenzoic acid, kaempferol, procyanidin B1, and B2. Moreover, a negative correlation was observed between PC1 and the protocatechuic. High component loadings for component 1 (PC1) were contributed by finger millet Zimbabwe (FMZM) variety. Epicatechin was the last phenolic compound to appear at the bottom of the plot. However, principal component 2 (PC2) inversely correlated with most of the phenolic compounds with the exception of the protocatechuic acid. Principal component analysis showed that most of the phenolic compounds in the millet varieties had clustered on the principal component 1 (PC1) axis, which may indicate homogeneity among the investigated varieties. Finger millet varieties were seen as the main contributor to the clustering on the right side of the cluster confirming that they contain most of the phenolic compounds.

**TABLE 3:** Principal component analysis of the phenolic compounds showing their percentage contribution to the total variations

	PC 1	PC 2	PC 3
Protocatechuic acid	0.02453	−0.02285	−0.00366
Catechin	0.68074	0.53444	0.28301
Epicatechin	0.12519	−0.59342	0.77301
Tryptophan	−0.04816	0.46168	0.39881
p‐hydroxybenzoic acid	0.01682	0.09318	−0.15252
Procyanidin B1	0.03317	−0.02493	−0.01318
Procyanidin B2	0.01726	0.01875	−0.05059
Kaempferol glycoside	−0.71855	0.37269	0.37054
Eigenvalue	1.64066	1.00370	0.90270
% variance	99.8990	0.05817	0.04265
Cumulative %	99.8990	99.9140	100

**FIGURE 4:**
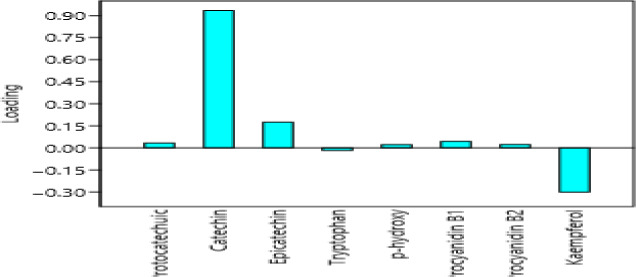
Loading plot of the phenolic compounds and their percentage contribution to the total variations

As shown in Table 3, the principal component1 (PC1) had the highest eigenvalue of 1.64066, which explains the highest variance of 99.899% in the principal component1 (PC1). As stated in Hair, Andrson, Tatham, and Black (1998), the eigenvalue greater than 1 is significant and a component loading which is more than ±0.3 is considered meaningful. Based on that information, this study attributes the variations to be caused by only a few phenolic compounds. In the case of the principal component 1 (PC1), the variation was influenced by catechin and kaempferol glycoside, which had a negative loading as shown in Figure 4. The major contributors to variation in the principal component 2 (PC2) are catechin, epicatechin, tryptophan, and kaempferol glycoside, and epicatechin had a negative loading. Principal component number 3 (PC3) was influenced by epicatechin, tryptophan, and kaempferol glycoside. It is noticeable that kaempferol glycoside contributed significantly in the variations of all the three components.

### 3.4 Pearson correlation (r) between the phenolic compounds

**FIGURE 5:**
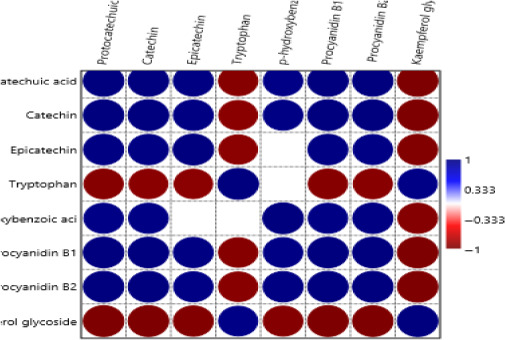
Pearson correlation plot for the phenolic compounds in the millet varieties

Pearson correlation was performed to investigate the correlations among the individual phenolic compounds (Figure 5). The results indicate that strong positive correlation exists between protocatechuic acid and catechin, epicatechin, p‐hydroxybenzoic acid, procyanidin B1, and B2. Exception was observed between protocatechuic acid and both tryptophan and kaempferol where a strong negative correlation existed. The same results were observed for catechin and epicatechin. There was also strong correlation between p‐hydroxybenzoic acid strongly correlated positively with procyanidin B1 and B2 and strong negative correlation with kaempferol. Pearson correlation plot in Figure 5 showed a significant negative correlation between p‐hydroxybenzoic acid and both epicatechin and tryptophan as indicated by the blank space in the figure. A general positive correlation among the phenolic compounds is evident as indicated by the blue circles.

### 3.5 Unknown compounds

**FIGURE 6:**
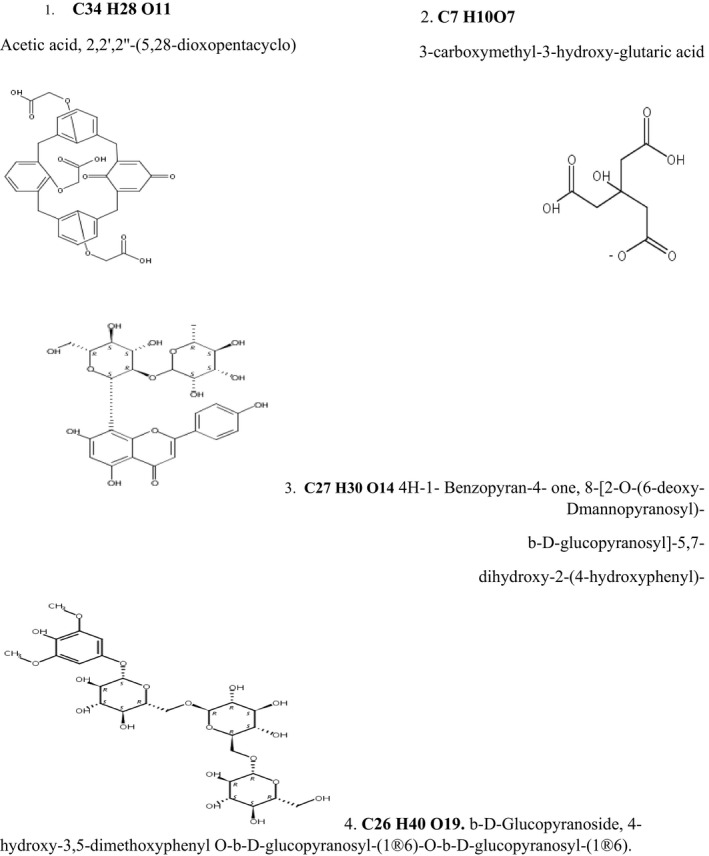
Possible fragmentation structures for compounds

A total of 4 unknown compounds with identified elemental formulas were also detected during the analysis. These compounds as presented in Figure 6 were assigned to possible compounds using SCI‐FINDER®. They include acetic acid, 2,2',2''‐(5.28‐dioxopentacyclo), glutaric acid, benzopyran, and b‐d‐glucopyranoside.

## 4 CONCLUSION

This study reports the profile of the phenolic compounds in the millet grains and confirms that the LC‐MS‐based profiling is a good technique for the phenolic characterization. It also substantiates the fact the millet grains are good source of nutrition and also beneficial for the health status of those who rely on it as staple food, due to the presence of nutraceutical components such as antioxidants and polyphenols. Because millet flavonoids play important roles in the prevention and management of type 2 diabetes, the finger millet varieties fit perfectly in terms of health benefits, as it contains the flavonoids more than the pearl millet types. The results of this study confirm that the type of millet was the determining factor of the phenolic contents. These results have provided useful information for effective utilization of millets as functional food ingredients for promoting health. Broader profiling of different millet varieties to include most of the varieties present in the Southern African region is encouraged.

The results of the current study demonstrated difference among the varieties of millet rather than the environmental effects, since finger millet varieties had more phenolic compounds than the pearl millet varieties regardless of the area of cultivation.

## CONFLICT OF INTEREST

The authors declare that there is no conflict of interest.

## ETHICAL APPROVAL

This study does not involve any human or animal testing.
